# Effect of rapamycin on lysosomal accumulation in a CRISPR/Cas9‐based cellular model of VPS13A deficiency

**DOI:** 10.1111/jcmm.17768

**Published:** 2023-05-10

**Authors:** A. R. Tornero‐Écija, M. A. Navas, S. Muñoz‐Braceras, O. Vincent, R. Escalante

**Affiliations:** ^1^ Instituto de Investigaciones Biomédicas “Alberto Sols”, CSIC‐UAM Madrid Spain; ^2^ Departamento de Bioquímica y Biología Molecular, Facultad de Medicina Universidad Complutense de Madrid Madrid Spain

**Keywords:** autophagy, chorea‐acanthocytosis, CRISPR/Cas9, rapamycin, VPS13A

## Abstract

VPS13A is a lipid transfer protein localized at different membrane contact sites between organelles, and mutations in the corresponding gene produce a rare neurodegenerative disease called chorea‐acanthocytosis (ChAc). Previous studies showed that VPS13A depletion in HeLa cells results in an accumulation of endosomal and lysosomal markers, suggesting a defect in lysosomal degradation capacity leading to partial autophagic dysfunction. Our goal was to determine whether compounds that modulate the endo‐lysosomal pathway could be beneficial in the treatment of ChAc. To test this hypothesis, we first generated a KO model using CRISPR/Cas9 to study the consequences of the absence of VPS13A in HeLa cells. We found that inactivation of VPS13A impairs cell growth, which precludes the use of isolated clones due to the undesirable selection of edited clones with residual protein expression. Therefore, we optimized the use of pool cells obtained shortly after transfection with CRISPR/Cas9 components. These cells are a mixture of wild‐type and edited cells that allow a comparative analysis of phenotypes and avoids the selection of clones with residual level of VPS13A expression after long‐term growth. Consistent with previous observations by siRNA inactivation, VPS13A inactivation by CRISPR/Cas9 resulted in accumulation of the endo‐lysosomal markers RAB7A and LAMP1. Notably, we observed that rapamycin partially suppressed the difference in lysosome accumulation between VPS13A KO and WT cells, suggesting that modulation of the autophagic and lysosomal pathway could be a therapeutic target in the treatment of ChAc.

## 1 INTRODUCTION

VPS13A is a lipid transfer protein localized at different membrane contact sites between organelles,^1^, ^2^, ^3^, ^4^, ^5^, ^6^ and mutations in the corresponding gene produce a rare neurodegenerative disease called chorea‐acanthocytosis (ChAc), also known as VPS13A disease.^7^, ^8^, ^9^ Several lines of evidence point to the involvement of the autophagic‐lysosomal pathway in ChAc. The first description of a possible link between the VPS13 family of proteins and the autophagic/lysosomal pathway comes from the study of TipC, a member of the VPS13 family in the model organism *Dictyostelium discoideum*. Inactivation of TipC leads to decreased autophagy flux and abnormal development in this organism.^10^ Consistent with this hypothesis, delayed digestion of phagosomal contents is observed in *Tetrahymena thermophila* mutant cells lacking a VPS13A homologue, leading to impaired phagocytosis.^11^ Furthermore, accumulation of p62 and protein aggregates is observed in the central nervous system of *Drosophila* lacking the VPS13A functional homologue.^12^ This hypothesis has been reinforced by studies of human red blood cells and reticulocytes from ChAc patients, which showed organelle remains and delayed clearance of mitochondria and lysosomes, respectively.^13^ Furthermore, Vps13a^−/−^ mice showed impaired autophagy in the basal ganglia with accumulation of toxic levels of the active kinase Lyn.^14^ All these results suggest that the autophagic/lysosomal pathway could constitute a potential therapeutic target for the treatment of ChAc.

The availability of simple cellular models is essential to identify compounds of therapeutic interest. Previous studies showed that VPS13A depletion in HeLa cells results in deficient lysosomal‐dependent degradation of autophagic and endocytic cargos, such as p62, LC3 and EGFR.^5^, ^10^ A partial defect in the maturation of the lysosomal hydrolase cathepsinB was also described.^5^ This impaired degradative capacity of lysosomes affects autophagy and endocytic degradation at a late stage, resulting in the perinuclear accumulation of vesicles containing partially degraded material (endolysosomes) as determined previously by electronic microscopy.^5^ This phenotype can be easily monitored by immunofluorescence analysis of endosomal and lysosomal markers.^5^, ^10^ Our goal was to optimize a HeLa cell model of VPS13A dysfunction based on these previous observations to determine, as a proof of concept, whether compounds that modulate the endo‐lysosomal pathway could be beneficial in the treatment of ChAc. To test this hypothesis, we use pooled cells obtained shortly after transfection with CRISPR/Cas9 components that generate a mixture of wild‐type and VPS13A‐edited cells, allowing a comparative analysis of phenotypes and avoiding the selection of clones with residual levels of VPS13A. Consistent with previous results obtained by siRNA treatment, VPS13A inactivation by CRISPR/Cas9 results in accumulation of the endo‐lysosomal marker LAMP1.^5^ We found that rapamycin, a potent activator of the autophagic/lysosomal pathway, compensates this cellular phenotype.

## 2 MATERIALS AND METHODS

### 2.1 HeLa cell culture and transfection

HeLa cells were a gift from Dr. Alberto Muñoz (Instituto de Investigaciones Biomédicas Alberto Sols). Cells were grown in complete DMEM medium (Dulbecco's modified Eagle's medium; Sigma‐Aldrich, D5648), supplemented with 10% FBS (foetal bovine serum; Gibco, 1027‐106) and 1X penicillin–streptomycin (Gibco, 15,140‐122).

Transfection of plasmid DNA was performed using Lipofectamine 2000 (Invitrogen, 11668019) according to the manufacturer's instructions. Briefly, Lipofectamine 2000 and DNA were mixed in serum‐reduced Opti‐MEM medium (Life Technologies, 31985062), and the DNA‐Lipofectamine complexes were added to the cells. The medium was changed to DMEM at 6 h after transfection.

Transfection of siRNAs has been performed as described previously,^5^ using siRNA s23340 (Ambion) for VPS13A inhibition, s29543 (Ambion) for VPS13C inhibition and 2‐4390846 (Ambion) as a siRNA control.

### 2.2 Plasmids

The guide RNA expression plasmid for CRISPR/Cas9 was obtained by annealing of two complementary oligonucleotides (F: 5′‐ CACCGTTCTTGGGGGACTATGTGG ‐3′; R: 5′‐ AAACCCACATAGTCCCCCAAGAAC) and cloning in the BsmB1 site of the BPK1520 vector (Addgene, #65777). The plasmid for Cas9 expression was eSPCas9 (Addgene, #71814). TFEB_WT‐MYC (Addgene, #99955).

### 2.3 CRISPR/Cas9 techniques

Three guide RNA expression plasmids were tested in T7 endonuclease 1 mismatch detection assays^15^ and the one that gave the best result was selected. For clonal isolation, the Cas9 and guide RNA expression plasmids were transfected in HeLa cells with Lipofectamine 2000 according to manufacturer's instructions. After 48 h post‐transfection, cells were disaggregated and serial dilutions were performed up to the limit dilution of 5 cells/mL, and 100 μL (0.5 cells/well) was cultured in 96‐well plates (Falcon, 353072). Optical microscopy was used to monitor the appearance of single growth foci (clones). The identified clones were disaggregated and plated in a new 96‐well plate (pass 1). When the cells reached 80%–90% confluence, they were transferred to a 24‐well plate (pass 2) and then to a 6‐well plate (pass 3). When they were again confluent, they were disaggregated and resuspended in 1 mL of fresh medium (DMEM). Part of these cells were left for further growth and study, while the remainder was washed with 1X PBS and used for genomic DNA extraction with the NZY Tissue gDNA isolation kit (Nzytech). The following oligonucleotides were used for amplification of the 348 base pairs (bp) genomic sequence containing the CRISPR/Cas9‐modified VPS13A sequence: F: 5′‐ CCGGTGAACCGAATTACCTC ‐3′; R: 5′‐ GCTTTACGTAACTTGACCCACAG −3′.

To obtain the pooled VPS13A‐CRISPR/Cas9 cells, Cas9 and guide RNA plasmids were transfected into HeLa cells previously deposited on coverslips using Lipofectamine 2000 according to the manufacturer's instructions, and the medium was changed after 6 h. Cells were maintained in culture during 6 days after transfection, with medium changed every 48 h. Cells were then fixed for immunofluorescence as described below. This time point after transfection was previously selected in an initial set‐up experiment by analysing the VPS13A expression levels by Western blot (WB) at different days after transfection (not shown). A decrease in expression level was clearly observed at 6 days after transfection.

### 2.4 Immunofluorescence analysis

Cells were seeded on sterile 12 × 12 mm glass coverslips, placed in 24‐well plates. They were fixed with 3% PFA (paraformaldehyde) in PBS (133 mM NaCl, 8 mM Na2HPO4, pH 7.4), for 30 min at room temperature (RT). Cells were washed with PBS and incubated with 100 mM glycine in PBS for 30 min at RT. Cells were then permeabilized with cold methanol at −20°C for 10 min. After washing with PBS, cells were incubated with blocking solution (3% Bovine serum albumin, 0.2% Triton X‐100 in PBS) for 1 h at RT. Subsequently, they were incubated for 3 h at RT with the primary antibodies dissolved in the blocking solution, washed with PBS, and incubated with the appropriate secondary antibodies in the blocking solution for 1 h at RT. Coverslips were washed with PBS and mounted on a slide using Prolong Diamond Antifade Mountant (Molecular probes, P36970). Images were acquired using an inverted laser confocal microscope (Zeiss, LSM710) and analysed with ImageJ software.

Antibodies used in this study: anti‐VPS13A (Sigma‐Aldrich, HPA021662) together with anti‐LAMP1 (Cell Signaling Technology, 15665), anti‐RAB7 (Cell Signaling Technology, 9367), anti‐EEA1 (BD Biosciences, 610456), anti‐MYC (Cell Signaling Technology, 2775). The LAMP1 signal ratio (A1/A2) is used as a measure of the level of accumulation. For this, cells were virtually divided into two halves and the area covered by the LAMP1 signal was measured using ImageJ (the larger signal was always assigned to A1).

### 2.5 Western‐blot (WB) analysis

For protein extraction, HeLa cells were washed with PBS and lysed for 1 h in lysis solution (50 mM Tris–HCL pH 8, 150 mM NaCl, 1% Triton X‐100), supplemented with protease inhibitors (Sigma‐Aldrich, P8340) (1:100) and phosphatase inhibitors (2.5 mM NaF, 0.2 mM Na3VO4). Cell lysates were centrifuged (12,000 g, 10 min, 4°C), and the concentration of protein present in the supernatant was measured using the commercial Pierce BCA Protein Assay Kit (Thermo Fisher Scientific, 23225). The protein sample was separated in NuPage Tris‐acetate 3%–8% polyacrylamide gradient gels (Invitrogen, EA0378). Proteins were transferred to PVDF membranes (Immobilon‐P polyvinylidene difluoride; Millipore, IPVH00010) by wet transfer. Subsequently, they were blocked in 5% milk powder in TBS‐T (50 mM Tris, 150 mM NaCl, 0.5% Tween) for 1 h at RT. The membranes were incubated with the anti‐VPS13A antibody dissolved in 5% milk powder in TBS‐T, at 4°C overnight. The next day, the membranes were washed with TBS‐T for 1 h and incubated with the corresponding secondary antibody dissolved in 2% milk powder in TBS‐T, at RT for 1 h. Finally, the membranes were washed again with TBS‐T for 1 h, before incubation with the chemiluminescent substrate (Super Signal West Pico PLUS Chemiluminescent substrate; Thermo Fisher, 34580). The chemiluminescent signal was detected using photographic films (CURIX RP2 Plus films; Agfa).

### 2.6 Rapamycin treatment

Cells were incubated with rapamycin (Calbiochem, 553210) at the indicated concentrations for 48 h before being fixed for immunofluorescence. For CRISPR/Cas9 VPS13A KO pool analysis, cells were treated with 100 nM rapamycin or equal volume of DMSO (dimethyl sulfoxide) 4 days after transfection with the guide RNA and Cas9 plasmids as described above. After 48 h of treatment, immunofluorescence was performed as described earlier.

### 2.7 Data and image analysis

Image analysis was performed with ImageJ software. Data obtained from different independent experiments were analysed with GraphPad Prism (GraphPad software). The mean of these data together with their standard deviation were plotted in different graphs with error bars. Mann–Whitney statistical analysis was used for comparison of two samples or conditions. For comparison of multiple conditions, Kruskal–Wallis statistical analysis was applied with Dunn's multiple comparison test. Each figure shows the analysis used and the *p*‐value obtained (*p < 0.05; **p < 0.01; ***p < 0.001; ****p < 0.0001).

## 3 RESULTS AND DISCUSSION

### 3.1 Clonal isolation of VPS13A edited HeLa cells using CRISPR/Cas9 results in the selection of clones with residual level of VPS13A expression

**FIGURE 1:**
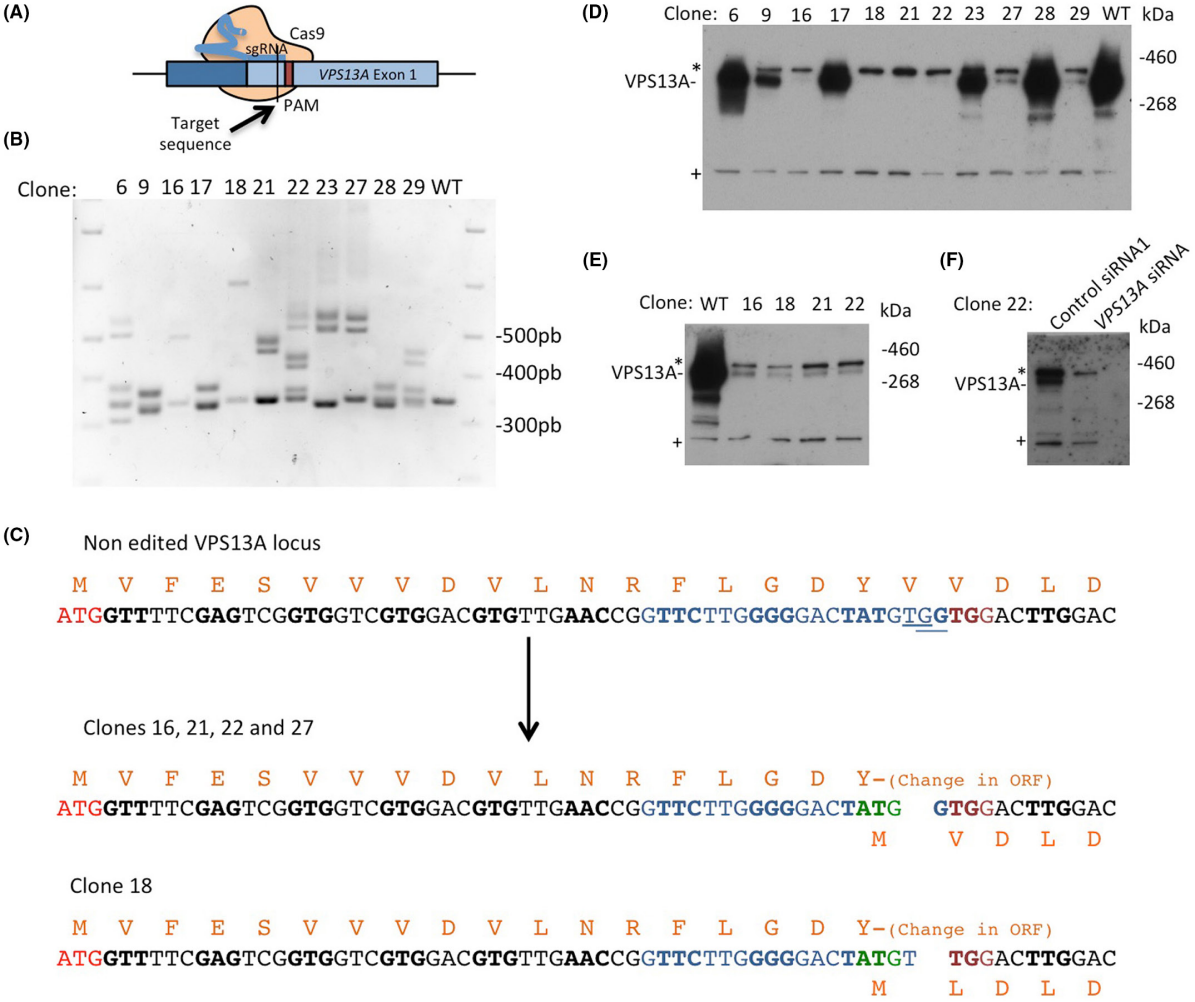
CRISPR/Cas9‐mediated editing of the VPS13A gene. (A) Representative schematic of the CRISPR/Cas9 method. The guide RNA (sgRNA) recognizes the target sequence located in exon 1 of VPS13A, allowing Cas9‐mediated cleavage of the DNA 3–4 base pairs (bp) upstream of the PAM sequence. (B) PCR amplification and agarose gel analysis of the genomic sequence flanking the sgRNA target. In WT cells, as expected a single 348 bp PCR product is detected while in the isolated clones, products of different size are observed, consistent with the presence of indels in one or more of the three alleles of VPS13A. Products of similar size to WT fragment were isolated and sequenced to ensure that they also correspond to edited alleles with small indels. (C) Genomic sequence corresponding to the N‐terminal region of VPS13A (non‐edited VPS13A locus) where the initiator codon ATG (red), the sgRNA target (blue) and the PAM sequence (dark red) are indicated. Codons are alternated in bold or normal font to more clearly delineate the protein open reading frame (ORF), and the corresponding amino acids are indicated above the codons. The underlined bases indicate the deletion generated in clones 16, 21, 22 and 27 (deletion of the 2 bp TG) and clone 18 (deletion of the 2 bp GG). The consequence of these deletions on the reading frame is shown below the arrow. Although these deletions disrupt the ORF, they result in an alternative initiation codon (green) that could allow the expression of an N‐terminal truncated protein. (D) Expression levels of VPS13A (expected size: 360 kDa) were analysed by WB (NuPAGE) in clones with ORF‐disrupting deletions. Clones 16, 18, 21 and 22 were selected for study due to the apparent absence of protein expression. The asterisks indicate a cross‐reacting band corresponding to VPS13C (Figure S1A), and the symbol + marks a non‐specific unidentified band. (E) VPS13A expression levels were reanalyzed in clones 16, 18, 21 and 22 after 15 days of growth. A residual level of VPS13A was detected. (F) The use of a siRNA specific for VPS13A in clone 22 resulted in the disappearance of the residual band, confirming the existence of residual levels of VPS13A in the isolated clones.

Our first objective was to isolate VPS13A KO clones in HeLa cells using the CRISPR/Cas9 technique. A guide sgRNA targeting a sequence located in exon 1 was designed (Figure 1A). Several clones were isolated by limiting dilution after transfection with the CRISPR/Cas9 plasmids. Genomic DNA was prepared from 11 clones, and PCR was performed using oligonucleotides flanking the target (Figure 1B). While a single PCR product of the expected size (348 bp) was detected in HeLa WT cells, all clones tested yielded additional bands of larger size, indicative of large insertions in some of the 3 alleles of the *VPS13A* gene in HeLa cells (Figure 1B). In addition, a PCR product migrating close to WT size was also detected in most clones. To verify that the corresponding allele also contained a deletion/insertion, this band was isolated and DNA was sequenced for 10 of the 11 clones. Half of the clones (6, 9, 17, 23, 28) contained a short deletion of 3, 6 or 12 base pair that maintained the open reading frame. In contrast, the other half (16, 18, 21, 22, 27) contained a two base pair deletion that disrupted the reading frame, with a one base shift in clone 18 (Figure 1C). Clones 6, 9, 17, 23 and 28, in which the reading frame of *VPS13A* was maintained, expressed medium or high levels of VPS13A protein, as detected by WB, whereas very weak or almost undetectable expression of VPS13A was observed in clones 16, 18, 21, 22 and 27, which had two base pair deletions (Figure 1D). The specificity of the VPS13A antibody in WB (Figure S1A) was assessed using protein extracts of VPS13A and/or VPS13C siRNA‐depleted HeLa cells. Note that the upper band corresponds to the cross‐reaction with VPS13C, but the size difference allows unambiguous determination of the VPS13A‐specific signal. We then selected the four clones (clones 16, 18, 21 and 22) that lacked detectable levels of VPS13A for further characterization (Figure 1D). In these clones, we initially observed a marked growth defect that progressively recovered with each passage. After 15 days of growth, we rechecked the level of VPS13A by WB and, surprisingly, found low but detectable levels of VPS13A (Figure 1E), as demonstrated by its sensitivity to siRNA treatment (Figure 1F). These observations indicate that the isolated clones still had detectable levels of VPS13A expression despite successful editing of all three alleles.

Interestingly, although the resulting deletions of clones 16, 18, 21, 22 disrupt the reading frame, their editing also generates a potential alternative initiation codon in phase with the *VPS13A* ORF that would result in a truncated protein lacking the first 19 amino acids (Figure 1C). Our results suggest that loss of VPS13A in HeLa cells impairs growth, which consequently favours the selection of clones that, although fully edited, have a residual level of VPS13A expression. The presence of residual levels of functional proteins in fully edited clones following CRISPR/Cas9 has been previously described and has been found to be more frequent than expected.^16^ To avoid this, we set out to optimize a CRISPR/Cas9‐based method for VPS13A that would eliminate the clonal selection step.

### 3.2 A CRISPR/Cas9‐based cell pool technique enables detection of accumulated late endosomes/lysosomes in VPS13A‐deficient HeLa cells

**FIGURE 2:**
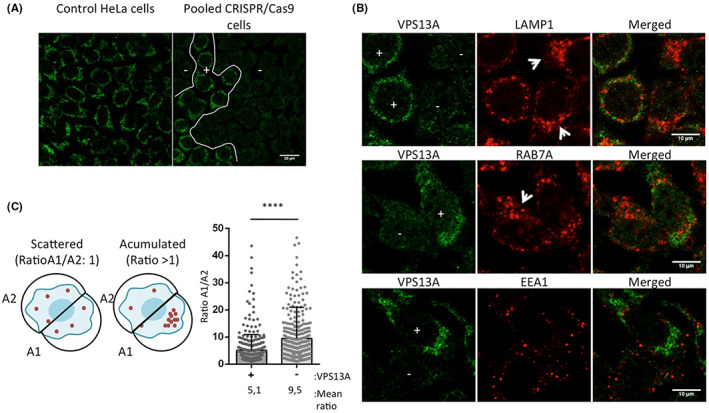
CRISPR/Cas9‐based HeLa model for VPS13A deficiency. (A) Pooled cells after transfection with the CRISPR/Cas9 plasmids contain a mixed population of edited and non‐edited cells suitable for further studies. HeLa cells were transfected with the CRISPR/Cas9 plasmids and maintained for 6 days under growth conditions before the analysis by immunofluorescence with the VPS13A antibody. Clusters containing VPS13A‐expressing cells (+) are mixed with groups containing non‐expressing cells (−). (B) VPS13A expression levels were analysed by immunofluorescence with the anti‐VPS13A antibody after 6 days of transfection with the CRISPR/Cas9 system elements. A population composed of cells expressing VPS13A (VPS13A+) and cells with no detectable levels of VPS13A (VPS13A−) was observed, likely representing a mixed population of WT and KO cells, respectively. The same cells were used for simultaneous detection of VPS13A with LAMP1, RAB7A or EEA1. Increased accumulation of LAMP1 and RAB7A was observed in VPS13A− cells (white arrows). (C) Quantification of the level of LAMP1 accumulation in VPS13A+ and VPS13A− cells. A simplified cartoon on the left shows scattered (non‐accumulated) distribution of the marker, and accumulated distribution of the marker. The area of LAMP1 signal was measured, and the ratio (A1/A2) is used as a measurement of the level of accumulation. On the right, a quantification of the A1/A2 ratio of the LAMP1 marker in VPS13A+ and VPS13A− cells is shown. Data were obtained from three independent transfections with the CRISPR/Cas9 plasmids and a total of 699 cells were analysed.

We reasoned that pooled cells taken shortly after transfection with CRISPR/Cas9 plasmids would contain a mixture of WT and edited cells not subjected to the selective long‐term growth pressure required for clonal isolation. As expected, clusters of VPS13A positive cells were intermixed with non‐expressing cells 6 days after transfection with the CRISPR/cas9 plasmids (Figure 2A). No such expression heterogeneity was observed in non‐transfected HeLa cells, indicating that the lack of expression is likely due to CRISPR/cas9‐derived gene editing. Detection of VPS13A by immunofluorescence in this pooled population allowed us to discriminate cells expressing VPS13A (VPS13A+) from edited cells with undetectable VPS13A expression (VPS13A−). The specificity of the antibody in immunofluorescence was assessed using VPS13A or VPS13C siRNA‐depleted cells (Figure S1B). Under these conditions, the antibody was highly specific for VPS13A, as the signal obtained in VPS13A‐depleted cells was almost undetectable, and the fluorescence pattern and intensity were not affected by VPS13C depletion. A limitation of this technique is that cells with low levels of VPS13A that might result from one unedited allele or from indels that maintain the reading frame might not give sufficient signal on immunofluorescence, and thus be classified as VPS13A−. Therefore, in addition to cells with complete absence of VPS13A, the VPS13A− pool may contain cells with low levels of VPS13A below the level of detection by immunofluorescence.

We performed simultaneous detection of endosomal markers and VPS13A to compare the distribution of these markers in VPS13A+ and VPS13A− subpopulations in the same microscopic preparation (Figure 2B). Most VPS13A− cells showed some level of accumulation of late endosomes (Rab7A) and lysosomes (LAMP1), whereas the early endosome marker EEA1 showed a scattered distribution in both VPS13A+ and VPS13A− cells (Figure 2B). These results are in agreement with previous data using VPS13A siRNA depletion in HeLa cells.^5^, ^10^ Late endosomes and autophagosomes move toward the perinuclear region as they mature to fuse with lysosomes that normally reside in this region.^17^ The increased perinuclear accumulation of late endosomal markers in VPS13A‐depleted HeLa cells suggests a defect in a late stage of degradation and/or recycling.^5^ This phenotype is thus a good readout of defects in the late stage of endolysosomal degradation. For quantification of this phenotype, cells were initially classified into two groups according to the presence of the VPS13A signal (VPS13A+ and VPS13A−). The cells were then virtually divided into two halves, and the ratio of the signal of the lysosomal marker LAMP1 in each half was used as a measure of the asymmetric distribution of this marker (Figure 2C). As expected, we observed an increased LAMP1 accumulation ratio in VPS13A− compared to VPS13A+ cells, which is consistent with the previously described phenotype.^5^

### 3.3 The autophagic/lysosomal pathway may be a potential therapeutic target in ChAc

**FIGURE 3:**
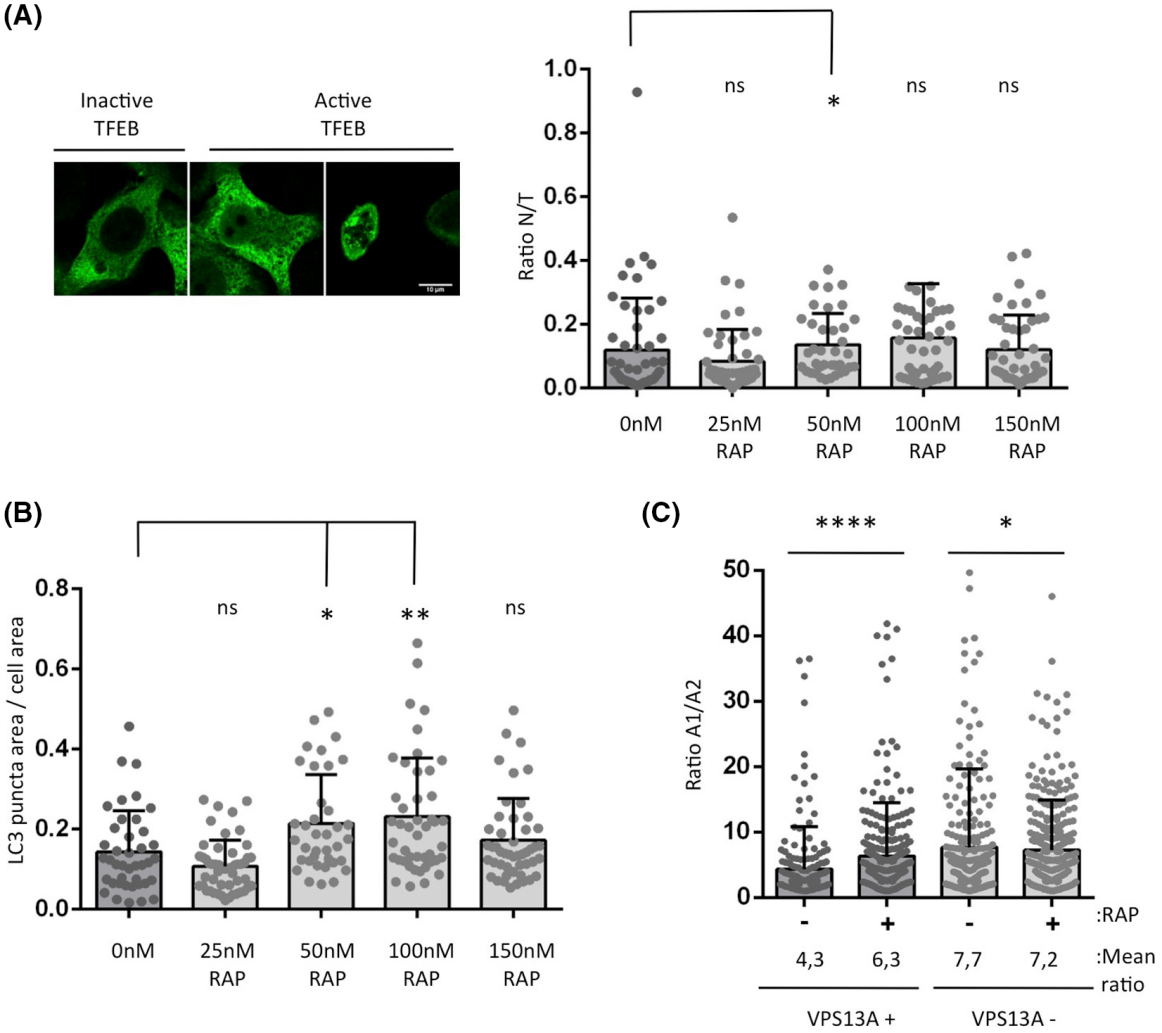
Rapamycin partially compensates for the lysosomal accumulation in VPS13A KO HeLa cells. (A) HeLa WT cells were transfected with the TFEB‐WT‐MYC plasmid and analysed by immunofluorescence with an anti‐MYC antibody. Left figure shows an example of cells without translocation of TFEB to the nucleus (inactive TFEB), and figures on the right show a partial or total translocation of the protein (active TFEB). The graph shows a quantitative analysis of TFEB activation using the ratio of the nuclear TFEB signal versus the total TFEB signal in the cell (N/T ratio), after treatment with different concentrations of rapamycin. Approximately 50 cells from one transfection were analysed for each concentration. Kruskal–Wallis statistical analysis was applied with Dunn's multiple comparison test (*p < 0.05; ns, not significant). (B) LC3 levels present in HeLa WT cells treated with different concentrations of rapamycin. The area occupied by LC3‐positive vesicles was normalized to the total cell area. Approximately 50 cells from one transfection were analysed for each concentration. Kruskal–Wallis statistical analysis was applied with Dunn's multiple comparison test (*p < 0.05, **p < 0.01; ns, not significant). (C) The A1/A2 ratio of the LAMP1 marker was quantified in VPS13A+ and VPS13A− cells treated with rapamycin (100 nM) or DMSO (as a control). Data are from three independent transfections in which 515 cells from DMSO control and 669 cells treated with rapamycin were analysed. Mann–Whitney statistical analysis was applied (*p < 0.05; **** *p* < 0.0001).

We hypothesized that the use of mTORC1 inhibitor rapamycin, a potent activator of lysosomal biogenesis and autophagy,^18^, ^19^, ^20^ could rescue this phenotype. Figure 3A,B shows a number of assays to determine the optimal concentration of rapamycin in HeLa cells that promotes translocation to the nucleus of the regulator of lysosomal biogenesis TFEB (Figure 3A), as well as upregulation of autophagic structures marked by LC3 (Figure 3B). From these set‐up experiments, 100 nM rapamycin was selected for further analysis. Pooled VPS13A‐CRISPR/Cas9 cells were treated with 100 nM rapamycin (or DMSO in the controls), fixed, and the accumulation ratio of LAMP1 in VPS13A+ and VPS13A− cells was compared (Figure 3C). In agreement with previous work showing that rapamycin induces lysosomal activity,^18^, ^19^, ^20^ we found an increased accumulation ratio of LAMP1 in rapamycin‐treated VPS13A+ cells. Because of the known defect of VPS13A‐depleted cells in the capacity of lysosomes to degrade autophagic/endosomal cargos, we would have expected a worsening of the phenotype in VPS13A− cells under rapamycin treatment due to increased autophagic cargo. In contrast, in VPS13A− cells, no further increase, but a slight decrease was observed, indicating that there is no cumulative effect of rapamycin treatment and VPS13A deficiency on LAMP1 accumulation. This result suggests that under these conditions, rapamycin‐dependent activation of lysosomal activity and autophagy partially compensates for the lysosomal dysfunction due to lack of VPS13A. However, it should be noted that rapamycin, through its inhibition of mTOR, may regulate pathways unrelated to lysosomal function, such as those related to insulin and amino acid signalling, which affect different aspects of cellular metabolism.^21^ We cannot rule out that other lysosome‐independent pathways may play an indirect role in the observed lysosomal phenotype. This potential effect of rapamycin suggests a possible therapeutic benefit that should be studied in more detail in other models closer to the disease, such as the mouse models developed by other research groups.^14^, ^22^, ^23^ Interestingly, rapamycin has been proposed as a possible treatment in other more prevalent neurodegenerative diseases such as Alzheimer's, Parkinson's and Huntington's diseases.

In summary, we have established a new simple cellular model of VPS13A deficiency based on CRISPR/cas9‐modified HeLa cells. Detailed characterization of CRISPR/Cas9 clones of VPS13A suggests that VPS13A inactivation impairs cell growth in this cell type, which generates a selective pressure to maintain residual levels of VPS13A protein in isolated clones. Very low levels of VPS13A appear to be sufficient to prevent the observed defects in lysosomal accumulation in this cell type. The unexpected sensitivity of HeLa cells to the lack of VPS13A is an experimental advantage that justifies the use of this model system to study VPS13A function but calls for caution when analysing phenotypes in isolated clones. To overcome the problem of clonal isolation, we use pooled cells after transfection, a convenient way to study subtle phenotypes due to the presence of an internal control, and which avoids the problem associated with studying genes that affect cell viability. Furthermore, this is the first time that rapamycin has been used in a preclinical study in the context of ChAc, and our results provide the first evidence suggesting that modulation of the autophagic/lysosomal pathway by rapamycin (or other TOR‐dependent pathways) may be beneficial. This finding warrants further investigation in more complex models on the potential use of rapamycin and the pathways affected by this treatment.

## AUTHOR CONTRIBUTIONS

**A.R. Tornero‐Écija:** Formal analysis (equal); investigation (equal); methodology (equal). **M.A. Navas:** Formal analysis (equal); investigation (equal); methodology (equal). **O. Vincent:** Conceptualization (equal); formal analysis (equal); funding acquisition (equal); project administration (equal); writing – review and editing (equal). **R. Escalante:** Conceptualization (equal); formal analysis (equal); funding acquisition (equal); project administration (equal); writing – original draft (lead); writing – review and editing (equal). **S. Muñoz‐Braceras:** Formal analysis (supporting); investigation (supporting); methodology (supporting).

## FUNDING INFORMATION

This work has been supported by the ‘Ministerio de Ciencia e Innovación’, grants numbers PGC2018‐093604‐B‐I00 (MICINN/AEI/FEDER, UE), PID2021‐127355OB‐I00 (MICIN/AEI/10.13039/501100011033/FEDER Una manera de hacer Europa). A.T. has been supported by Garantía Juvenil Program from Comunidad de Madrid and Advocacy for Neuroacanthocytosis patients (INT‐GB/0775).

## CONFLICT OF INTEREST STATEMENT

The authors declare no conflict of interests.

## Supporting information


Figure S1.

## Data Availability

The data that support the findings of this study are available from the corresponding author upon reasonable request.

## References

[1] Kumar N , Leonzino M , Hancock‐Cerutti W , et al. VPS13A and VPS13C are lipid transport proteins differentially localized at ER contact sites. J Cell Biol. 2018;217:3625‐3639. 30093493 10.1083/jcb.201807019 PMC6168267

[2] Leonzino M , Reinisch KM , De Camilli P . Insights into VPS13 properties and function reveal a new mechanism of eukaryotic lipid transport. Biochim Biophys Acta Mol Cell Biol Lipids. 2021;1866:159003. 34216812 10.1016/j.bbalip.2021.159003 PMC8325632

[3] Dziurdzik SK , Conibear E . The Vps13 family of lipid transporters and its role at membrane contact sites. Int J Mol Sci. 2021;22:2905. 33809364 10.3390/ijms22062905 PMC7999203

[4] Yeshaw WM , van der Zwaag M , Pinto F , et al. Human VPS13A is associated with multiple organelles and influences mitochondrial morphology and lipid droplet motility. Elife. 2019;8:e43561. 30741634 10.7554/eLife.43561 PMC6389287

[5] Muñoz‐Braceras S , Tornero‐Écija AR , Vincent O , Escalante R . VPS13A is closely associated with mitochondria and is required for efficient lysosomal degradation. Dis Model Mech. 2019;12:dmm036681. 30709847 10.1242/dmm.036681 PMC6398486

[6] Rzepnikowska W , Flis K , Muñoz‐Braceras S , Menezes R , Escalante R , Zoladek T . Yeast and other lower eukaryotic organisms for studies of Vps13 proteins in health and disease. Traffic. 2017;18:711‐719. 28846184 10.1111/tra.12523

[7] Peikert K , Danek A , Hermann A . Current state of knowledge in chorea‐Acanthocytosis as core Neuroacanthocytosis syndrome. Eur J Med Genet. 2018;61:699‐705. 29253590 10.1016/j.ejmg.2017.12.007

[8] Rampoldi L , Dobson‐Stone C , Rubio JP , et al. A conserved sorting‐associated protein is mutant in chorea‐acanthocytosis. Nat Genet. 2001;28:119‐120. 11381253 10.1038/88821

[9] Ueno S , Maruki Y , Nakamura M , et al. The gene encoding a newly discovered protein, chorein, is mutated in chorea‐acanthocytosis. Nat Genet. 2001;28:121‐122. 11381254 10.1038/88825

[10] Muñoz‐Braceras S , Calvo R , Escalante R . TipC and the chorea‐acanthocytosis protein VPS13A regulate autophagy in *Dictyostelium* and human HeLa cells. Autophagy. 2015;11:918‐927. 25996471 10.1080/15548627.2015.1034413 PMC4507429

[11] Samaranayake HS , Cowan AE , Klobutcher LA . Vacuolar protein sorting protein 13A, TtVPS13A, localizes to the *Tetrahymena thermophila* phagosome membrane and is required for efficient phagocytosis. Eukaryot Cell. 2011;10:1207‐1218. 21764909 10.1128/EC.05089-11 PMC3187053

[12] Vonk JJ , Yeshaw WM , Pinto F , et al. Drosophila Vps13 is required for protein homeostasis in the brain. PLoS One. 2017;12:e0170106. 28107480 10.1371/journal.pone.0170106 PMC5249141

[13] Lupo F , Tibaldi E , Matte A , et al. A new molecular link between defective autophagy and erythroid abnormalities in chorea‐acanthocytosis. Blood. 2016;128:2976‐2987. 27742708 10.1182/blood-2016-07-727321 PMC5179337

[14] Peikert K , Federti E , Matte A , et al. Therapeutic targeting of Lyn kinase to treat chorea‐acanthocytosis. Acta Neuropathol Commun. 2021;9:81. 33941276 10.1186/s40478-021-01181-y PMC8091687

[15] Mashal RD , Koontz J , Sklar J . Detection of mutations by cleavage of DNA heteroduplexes with bacteriophage resolvases. Nat Genet. 1995;9:177‐183. 7719346 10.1038/ng0295-177

[16] Smits AH , Ziebell F , Joberty G , et al. Biological plasticity rescues target activity in CRISPR knock outs. Nat Methods. 2019;16:1087‐1093. 31659326 10.1038/s41592-019-0614-5

[17] Pu J , Guardia CM , Keren‐Kaplan T , Bonifacino JS . Mechanisms and functions of lysosome positioning. J Cell Sci. 2016;129:4329‐4339. 27799357 10.1242/jcs.196287 PMC5201012

[18] Martina JA , Chen Y , Gucek M , Puertollano R . MTORC1 functions as a transcriptional regulator of autophagy by preventing nuclear transport of TFEB. Autophagy. 2012;8:903‐914. 22576015 10.4161/auto.19653 PMC3427256

[19] Noda T , Ohsumi Y . Tor, a phosphatidylinositol kinase homologue, controls autophagy in yeast. J Biol Chem. 1998;273:3963‐3966. 9461583 10.1074/jbc.273.7.3963

[20] Peña‐Llopis S , Brugarolas J . TFEB, a novel mTORC1 effector implicated in lysosome biogenesis, endocytosis and autophagy. Cell Cycle. 2011;10:3987‐3988. 22101272 10.4161/cc.10.23.18251 PMC3272281

[21] Zhu J , Wang H , Jiang X . mTORC1 beyond anabolic metabolism: regulation of cell death. J Cell Biol. 2022;221:e202208103. 36282248 10.1083/jcb.202208103 PMC9606688

[22] Sakimoto H , Nakamura M , Nagata O , Yokoyama I , Sano A . Phenotypic abnormalities in a chorea‐acanthocytosis mouse model are modulated by strain background. Biochem Biophys Res Commun. 2016;472:118‐124. 26921443 10.1016/j.bbrc.2016.02.077

[23] Tomemori Y , Ichiba M , Kusumoto A , et al. A gene‐targeted mouse model for chorea‐acanthocytosis. J Neurochem. 2005;92:759‐766. 15686477 10.1111/j.1471-4159.2004.02924.x

