# Supplementary figures and images for: Effect of rapamycin on lysosomal accumulation in a CRISPR/Cas9‐based cellular model of VPS13A deficiency
Source: J Cell Mol Med. 2023 May 10;27(11):1557–64. doi: 10.1111/jcmm.17768 (PMC10243151; doi:10.1111/jcmm.17768)

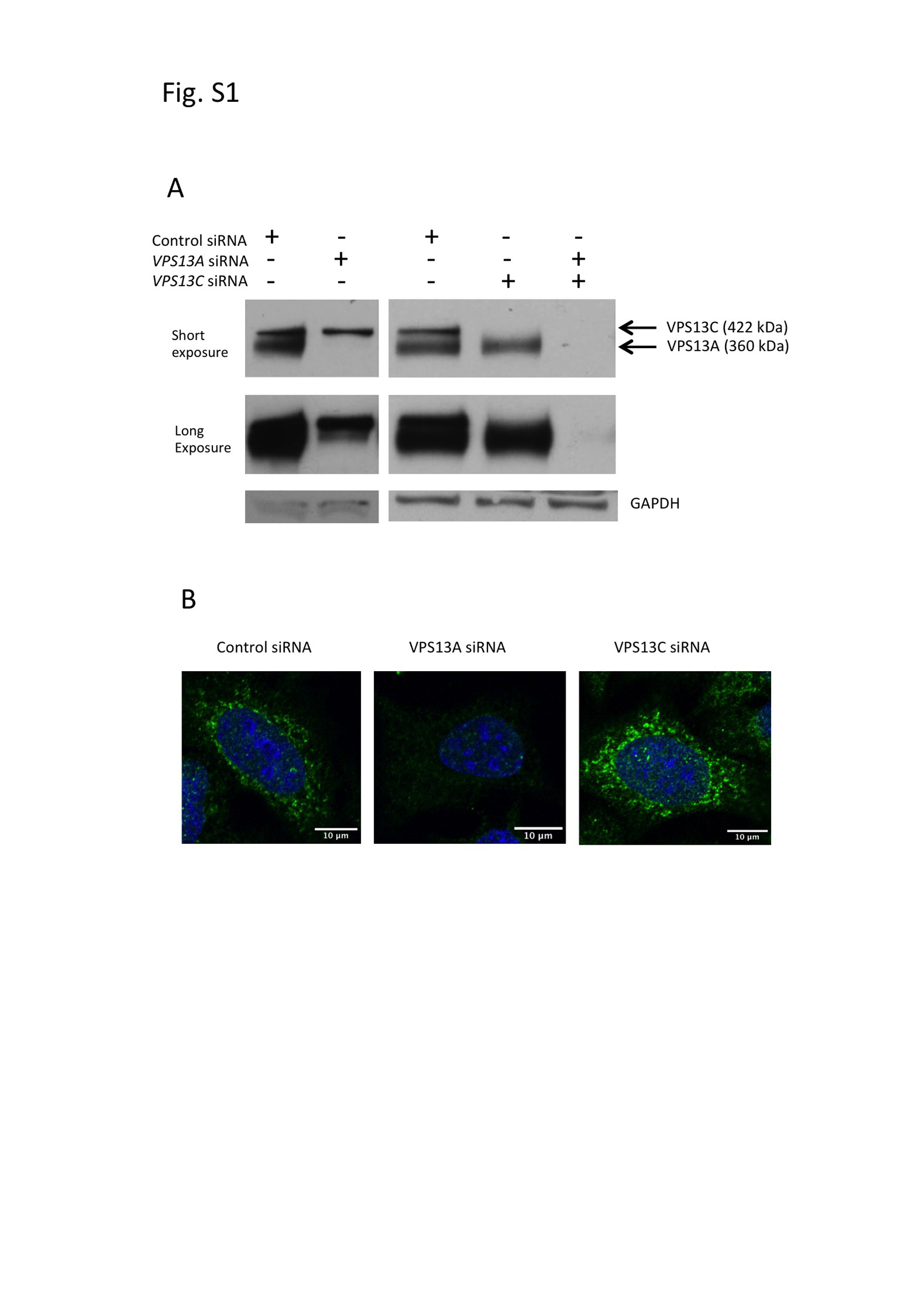

Supplement: Supplementary file 1 — Figure S1. [file JCMM-27-1557-s001.jpg]
